# Sleep duration and risk of post-stroke depression: a systematic review and meta-analysis

**DOI:** 10.3389/fneur.2026.1914895

**Published:** 2026-10-29

**Authors:** Mengyuan Li, Huihui Zhu, Furong Sui, Yican Tian, Wenyu Tang, Jingui Lai, Lu Wang, Lin Zhang, Yan Song

**Affiliations:** 1 School of Nursing, Beihua University, Jilin, China; 2 Department of Gastroenterology, Jilin City People’s Hospital, Jilin, China

**Keywords:** meta-analysis, post-stroke depression, sleep, sleep duration, stroke

## Abstract

**Background:**

Post-stroke depression frequently occurs as a complication following a stroke, with sleep duration identified as a risk factor. However, the relationship between sleep duration and the risk of post-stroke depression remains controversial.

**Objective:**

To investigate the association between sleep duration and the risk of post-stroke depression.

**Methods:**

Relevant studies investigating the association between sleep duration and post-stroke depression were searched in five English databases (PubMed, Cochrane Library, Embase, Web of Science, and CINAHL) and four Chinese databases (CNKI, Wanfang Data, VIP Database, and China Biology Medicine Database) from inception to January 2026. Data extraction and quality assessment of the included literature were independently performed by two investigators. Meta-analyses were conducted using Stata 16.0 software. Pooled odds ratios (ORs) and corresponding 95% confidence intervals (CIs) were calculated using either a fixed-effects or a random-effects model.

**Results:**

The analysis included 6 articles, encompassing a total of 5,624 cases. Meta-analysis showed that short sleep duration was associated with an increased risk of post-stroke depression [OR = 1.93, 95% CI(1.56–2.38), *p* < 0.05], whereas long sleep duration was not significantly associated with the risk [OR = 1.29, 95% CI(1.00–1.66), *p* > 0.05].

**Conclusion:**

Sleep duration is associated with the risk of post-stroke depression, with shorter sleep duration elevating this risk. Future studies should be incorporated to enhance the reliability of these findings.

**Systematic review registration:**

https://www.crd.york.ac.uk/PROSPERO/view/CRD42024609869, identifier CRD42024609869.

## 1 Introduction

Post-stroke depression (PSD) is an emotional-disorder syndrome that may emerge following a stroke, which is mainly characterized by low mood, loss of interest, and slow thinking (1), and it seriously affects patients’ quality of life (2). Clinical studies indicate that the incidence of PSD can reach up to 79% within 2 years following a stroke (3). When PSD occurs, disability and mortality rates can soar to 70–90% (4). Without timely detection and intervention, PSD not only severely hinders the recovery of neurological function and daily living abilities but also increases the burden on families and society. In extreme cases, it may even lead to suicidal behaviors (5).

The pathogenesis of post-stroke depression is complex (6), influenced by a variety of factors. Short or long sleep duration is a potential risk factor for post-stroke depression (7). The American Academy of Sleep Medicine (8) and the National Sleep Foundation (9) recommend that adults ideally sleep 7 to 9 h per day. Adequate sleep is essential for both physical and mental health, while both long (>9 h) and short sleep (<7 h) can heighten the risk of adverse health outcomes. Previous research has established a strong association between shorter or longer sleep duration and an increased risk of cardiovascular diseases, hypertension, hyperlipidemia, obesity, lung cancer, and other conditions (10–14). Recently, the association between sleep duration and post-stroke depression has garnered increasing attention (15). Xiao W (16) conducted a cross-sectional survey of 963 stroke patients, and found a significant association between sleep duration and depressive symptoms. In a South Korean cross-sectional study, stroke patients over 19 years old were examined (17), revealing that those with a sleep duration of ≤5 h faced a higher risk of post-stroke depression. The study by Dong L (18) identified an association between sleep duration of <6 h and post-stroke depression. Reis C (19) conducted a cross-sectional study involving 5,436 adults over 18 years old, finding that sleep duration of ≥9 h increased the risk of post-stroke depression. However, another cohort study of adults found no significant association between sleep duration of ≥9 h and post-stroke depression (20).

In summary, the association between sleep duration and the risk of post-stroke depression remains controversial. Therefore, we performed a meta-analysis of published studies concerning sleep duration and the risk of post-stroke depression to investigate the association between sleep duration and the risk of post-stroke depression, so as to provide theoretical evidence for the prevention of post-stroke depression.

## 2 Methods

### 2.1 Search strategy

Relevant studies investigating the association between sleep duration and post-stroke depression were searched in five English databases (PubMed, Cochrane Library, Embase, Web of Science, and CINAHL) and four Chinese databases (CNKI, Wanfang Data, VIP Database, and China Biology Medicine Database) from inception to January 2026. The search terms and search strategy are presented using PubMed as an example (see Table 1).

**Table 1:** Search strategy.

No	Search term
#1	(Sleep Duration) OR (Duration, Sleep) OR (Total Sleep Time) OR (Sleep Quantity) OR (Quantity, Sleep) OR (Sleep Quantities)
#2	(Stroke*) OR (Cerebrovascular Accident) OR (Cerebral Stroke) OR (Stroke, Cerebral) OR (Cerebrovascular Apoplexy) OR (Vascular Accident, Brain) OR (Brain Vascular Accident) OR (Vascular Accidents, Brain) OR (Cerebrovascular Stroke) OR (Apoplexy) OR (CVA (Cerebrovascular Accident)) OR (Acute Stroke) OR (Cerebrovascular Accident, Acute)
#3	(Depression) OR (Depressive Symptoms) OR (Depressive Symptom) OR (Symptom, Depressive) OR (Emotional Depression) OR (Depression, Emotional)
#4	#1 AND #2 AND #3

### 2.2 Inclusion and exclusion criteria

Inclusion criteria include: (1) Study types: Observational studies, such as cross-sectional, cohort, and case–control studies; (2) Study subjects: Individuals aged 18 years or older; (3) Outcome indicators: Post-stroke depression and poor mental health status; (4) Exposure factors: Sleep duration; and (5) Effect values must include at least one of the following: Relative risk (RR), odds ratio (OR), or hazard ratio (HR), along with their 95% confidence intervals (95%CI).

Exclusion criteria include: (1) Literature not in Chinese or English; (2) Review articles or case studies; and (3) Literature with missing or non-extractable data, or where the full text is unavailable.

### 2.3 Definition of sleep duration

The American Academy of Sleep Medicine (8) and the National Sleep Foundation (9) recommend that adults ideally sleep 7 to 9 h per day. However, definitions of short, long, and normal sleep duration vary across studies due to regional, cultural, and racial differences.

### 2.4 Data extraction and quality assessment

This study employed the retrieval strategy and analysis method developed by the Cochrane Collaboration (21) and conducted a systematic review following the Preferred Reporting Items for Systematic Reviews and Meta-Analyses (PRISMA statement) (22). The protocol had been registered with PROSPERO under the registration number CRD42024609869. Two researchers independently utilized Excel 2021 to analyze and organize the collected data. The extracted information included the first author, publication year, study area, sample size, sleep time assessment method, sleep time with its corresponding OR value and 95% CI, study type, post-stroke depression assessment method, covariates, Newcastle-Ottawa Scale (NOS) (23), and the Agency for Healthcare Research and Quality (AHRQ) (24) score. Cross-checks were performed by two individuals, and disputes were resolved through discussion or by consulting a third investigator. The NOS scale is used to assess cohort studies and comprises 8 items, with the “comparability between groups” item accounting for 2 points, while the remaining items each account for 1 point. Articles scoring 7 points or higher on the NOS are considered high-quality. The AHRQ scale is applied for cross-sectional studies and includes 11 items, answered with “Yes,” “No,” or “Unclear.” A score of 0 indicates “No” or “Unclear,” while 1 indicates “Yes.” Scores of 0 to 3, 4 to 7, and 8 to 11 define low, medium, and high quality, respectively.

### 2.5 Data analysis

Statistical analysis was conducted using Stata 16.0 software. In articles reporting multiple sleep time periods, we analyzed the effect sizes by combining the shortest and longest sleep duration from each study. For effect size selection, the odds ratio (OR) was employed for binary variables, all presented with 95% confidence intervals. Heterogeneity was assessed using the I^2^ statistic. An I^2^ value of less than 50% indicated low heterogeneity, and a fixed-effect model was used; otherwise, a random-effects model was applied. When necessary, sensitivity or subgroup analyses were performed to identify sources of heterogeneity. Additionally, subgroup analysis was conducted to investigate the impact of subgroups on effect size, specifically to determine if differences existed in the association between sleep duration and PSD across various subgroups. Sensitivity analysis involved excluding one or more studies based on specific bias considerations, followed by a re-conducted meta-analysis. The stability and reliability of the results were evaluated by observing changes in the combined analysis results and heterogeneity. Egger’s and Begg’s tests were used to assess publication bias (25). If the *p*-values for these tests were 0.05 or greater, it indicated no significant publication bias; otherwise, publication bias was present.

### 2.6 Differences between protocol and review

After completing the literature search, we found that restricting inclusion to prospective observational studies yielded too few eligible studies, resulting in an insufficient sample size and limited statistical power. To provide a more comprehensive analysis of the association between sleep duration and post-stroke depression, we therefore extended the inclusion criteria to also include high-quality cross-sectional studies. Accordingly, the original protocol criterion of “prospective observational studies” was revised to “observational studies.”

## 3 Results

### 3.1 Literature screening

Initially, 1,234 articles were retrieved from the databases. Following a step-by-step screening and thorough review, 6 articles were ultimately included in the meta-analysis. Figure 1 illustrates the detailed screening process and results.

**Figure 1:**
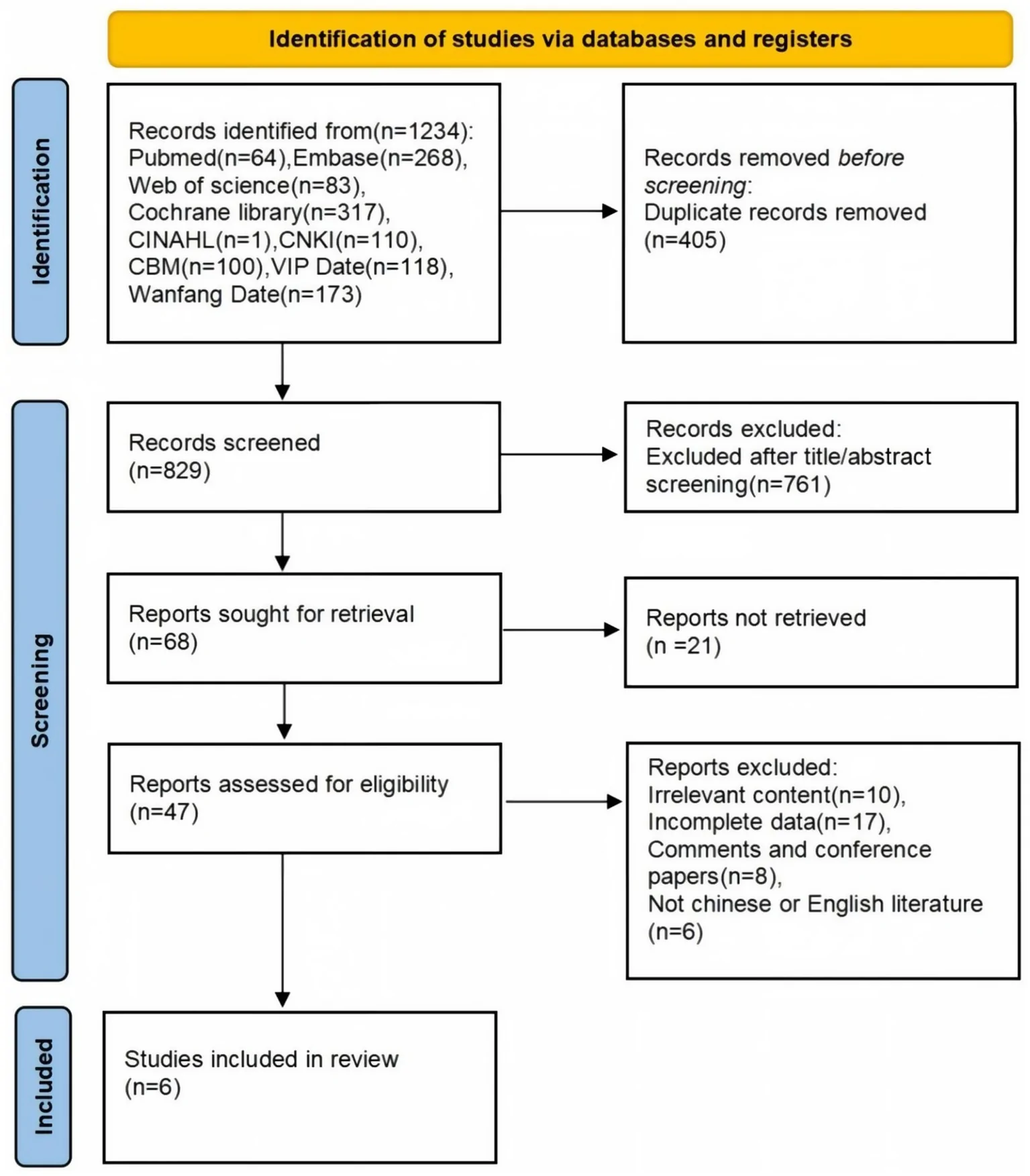
Flow chart of literature screening.

### 3.2 Quality evaluation of literature characteristics

Of the six included articles, two were from China, one from South Korea, and three from the United States, contributing to a total sample size of 5,624 cases. Two cohort studies were assessed using the Newcastle-Ottawa Scale (NOS), each receiving a score of 7 points, signifying high quality. Four cross-sectional studies were evaluated based on the Agency for Healthcare Research and Quality (AHRQ) criteria, with scores ranging from 7 to 9 points. Overall, the methodological quality of the included studies was good, as detailed in Table 2.

**Table 2:** Basic characteristics of the included studies.

Author	Year	Country	Sample size	Sleep duration assessment methods	Sleep duration (h/d)	OR (95% CI)	Study design	Outcome assessment	covariate	Study quality (AHRQ/NOS)
Liu (27)	2021	China	1,446	PSQI	<5	3.05 (1.85, 5.02)	Cohort study	PHQ-9	①②③⑥⑦⑧⑨⑩⑫⑭	7
					5–6	1.47 (1.00, 2.17)				
					6–7	0.92 (0.64, 1.30)				
					>7	Reference				
Chen (31)	2025	United States	1,101	Sleep Disorders Questionnaire	<7	2.19 (1.28, 3.75)	Cross-sectional study	PHQ-9	①②③④⑤⑦⑧⑨⑮	8
					7–8	Reference				
					>8	1.71 (0.84, 3.47)				
Dong (17)	2021	United States	1,369	Self-reported	<6	1.96 (1.38, 2.79)	Cohort study	PHQ-8	①②③④⑩⑫⑬⑮⑯	7
					6	1.10 (0.73, 1.67)				
					7–8	Reference				
					>8	1.08 (0.74, 1.57)				
Moon (16)	2019	Korea	504	Self-reported	≤5	1.88 (1.04, 3.42)	Cross-sectional study	Mental health items	①②③⑤⑥⑦⑧⑨⑪⑮	7
					6–8	Reference				
					≥9	1.56 (0.63, 3.87)				
Wang (35)	2021	China	836	Self-reported	<7	1.33 (0.70, 2.51)	Cross-sectional study	SDS HAMD-17	①②③⑦⑨⑧⑮	8
					7–8	Reference				
					8–9	0.98 (0.63, 1.53)				
					9–10	1.10 (0.69, 1.76)				
					≥10	1.50 (0.89, 2.53)				
Sawadogo (36)	2025	United States	368	Self-reported	≤6	0.76 (0.34, 1.71)	Cross-sectional study	12-Item Short Form Health Survey, Version 2	①②③④⑤ ⑥⑦⑧⑨⑮⑰⑱	9
					7–8	Reference				
					≥9	1.19 (0.52, 2.73)				

① Age, gender; ② marital status; ③ education; ④ race/ethnicity; ⑤ income; ⑥ employment status; ⑦ BMI; ⑧ smoking, alcohol use; ⑨ physical activity conditions; ⑩ functional status; ⑪ living alone, place of residence; ⑫ severity of stroke; ⑬ stroke type; ⑭ wards/specialties; ⑮ medical history; ⑯ Pre-stroke characteristics; ⑰ geographic region; ⑱ years since stroke diagnosis.

PHQ-9, Patient Health Questionnaire-9; PHQ-8, Patient Health Questionnaire-8; SDS, self rating depression scale; HAMD-17, Hamilton depression scale-1.

### 3.3 Meta-analysis

Six studies reported the effect of short sleep duration on the risk of post-stroke depression. The effect sizes for the shortest sleep duration in each study were pooled using a fixed-effect model. Heterogeneity was observed among the studies (I^2^ = 49.4%, *p* = 0.078). Short sleep duration was associated with an increased risk of post-stroke depression [OR = 1.93, 95% CI(1.56–2.38), *p* < 0.05], as illustrated in Figure 2.

**Figure 2:**
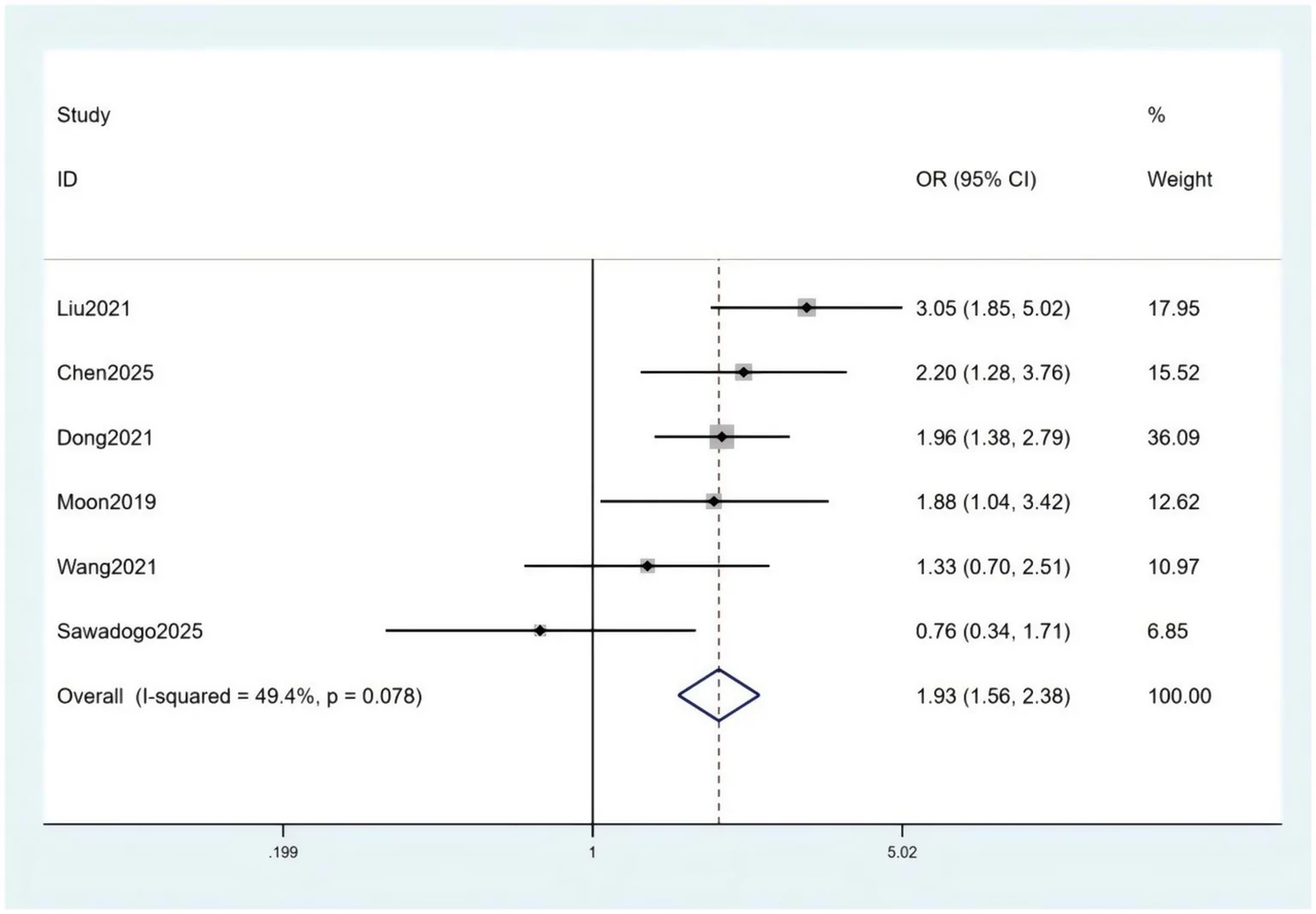
Forest plot of the association between short sleep duration and the risk of post-stroke depression.

Five studies reported the effect of long sleep duration on the risk of post-stroke depression. The effect sizes for the longest sleep duration in each study were pooled using a fixed-effect model. No heterogeneity was observed among the studies (I^2^ = 0.0%, *p* = 0.738). Long sleep duration was not associated with the risk of post-stroke depression [OR = 1.29, 95% CI(1.00–1.66), *p* > 0.05], as illustrated in Figure 3.

**Figure 3:**
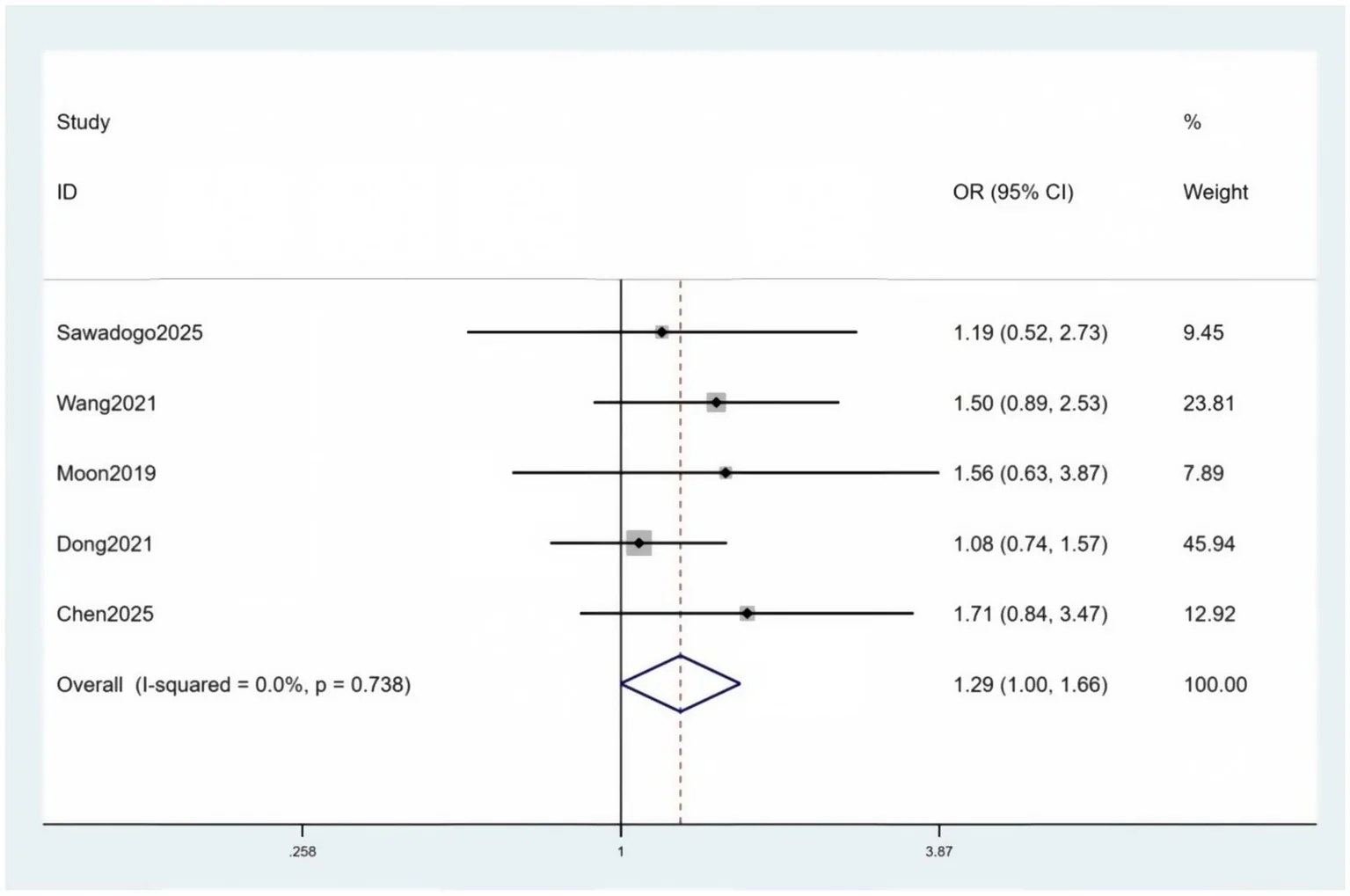
Forest plot of the association between long sleep duration and the risk of post-stroke depression.

### 3.4 Subgroup analysis

No significant association was observed between long sleep duration and the risk of post-stroke depression (PSD), and no heterogeneity was detected (I^2^ = 0.0%). Therefore, subgroup analyses were not performed for long sleep duration. For short sleep duration, subgroup analyses were conducted according to geographic region and study design.

Three studies reported the effect of short sleep duration on the risk of post-stroke depression in Asian populations. Heterogeneity was observed among these studies (I^2^ = 52.9%, *p* = 0.120). Short sleep duration was associated with an increased risk of post-stroke depression in Asian populations [OR = 2.11, 95% CI(1.52–2.94), *p* < 0.05]. Three studies reported the effect of short sleep duration on the risk of post-stroke depression in US populations. Heterogeneity was observed among these studies (I^2^ = 61.0%, *p* = 0.077). Short sleep duration was associated with an increased risk of post-stroke depression in US populations [OR = 1.81, 95% CI(1.37–2.38), *p* < 0.05], as illustrated in Figure 4.

**Figure 4:**
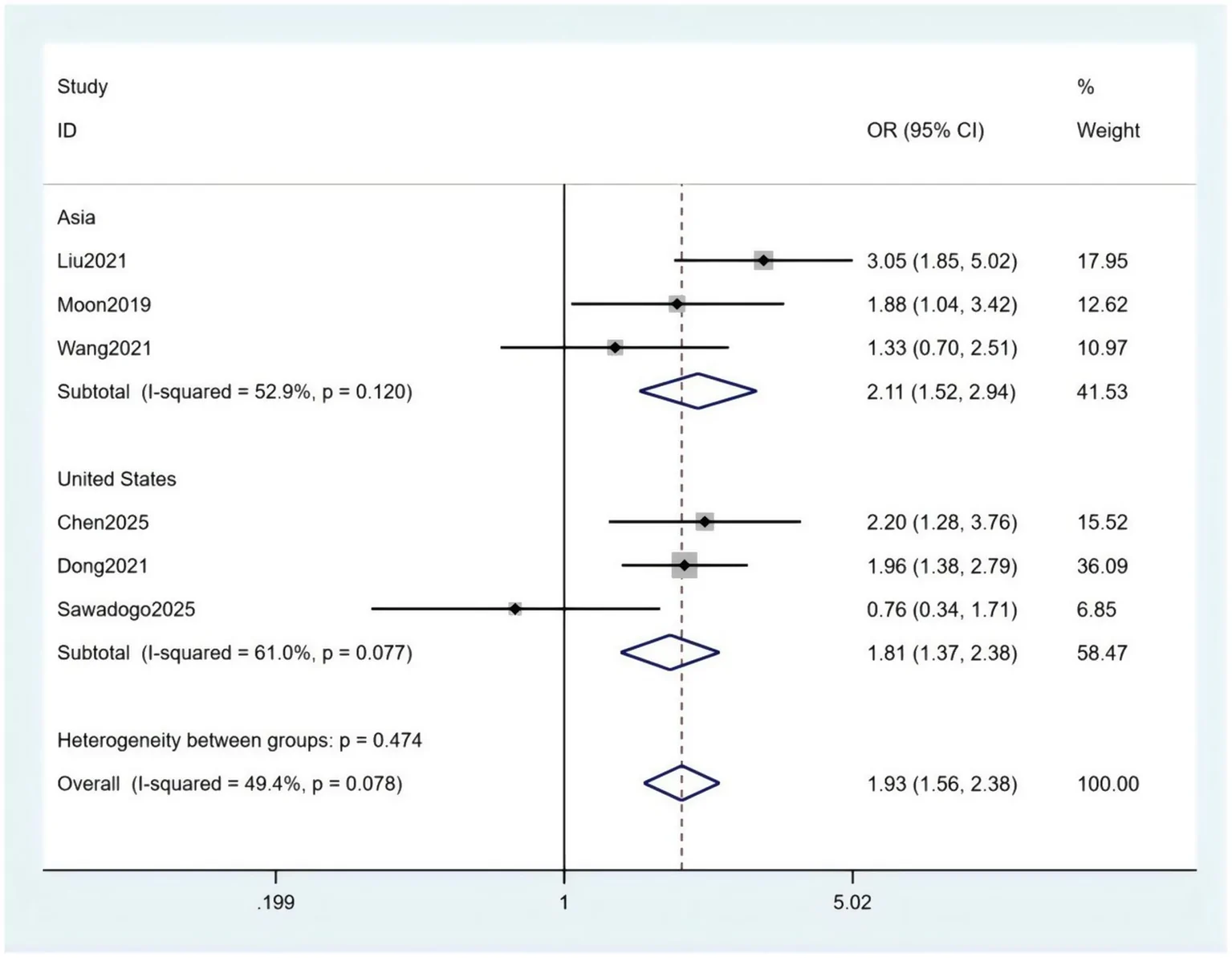
Forest plot of the association between short sleep duration and the risk of post-stroke depression in different geographic regions.

Two studies were cohort studies. Heterogeneity was observed among these studies (I^2^ = 50.5%, *p* = 0.156). In cohort studies, short sleep duration was associated with an increased risk of post-stroke depression [OR = 2.27, 95%CI(1.70–3.03), *p* < 0.05]. Four studies were cross-sectional studies. Heterogeneity was observed among these studies (I^2^ = 42.4%, *p* = 0.157). In cross-sectional studies, short sleep duration was associated with an increased risk of post-stroke depression [OR = 1.59, 95% CI(1.17–2.18), *p* < 0.05], as illustrated in Figure 5.

**Figure 5:**
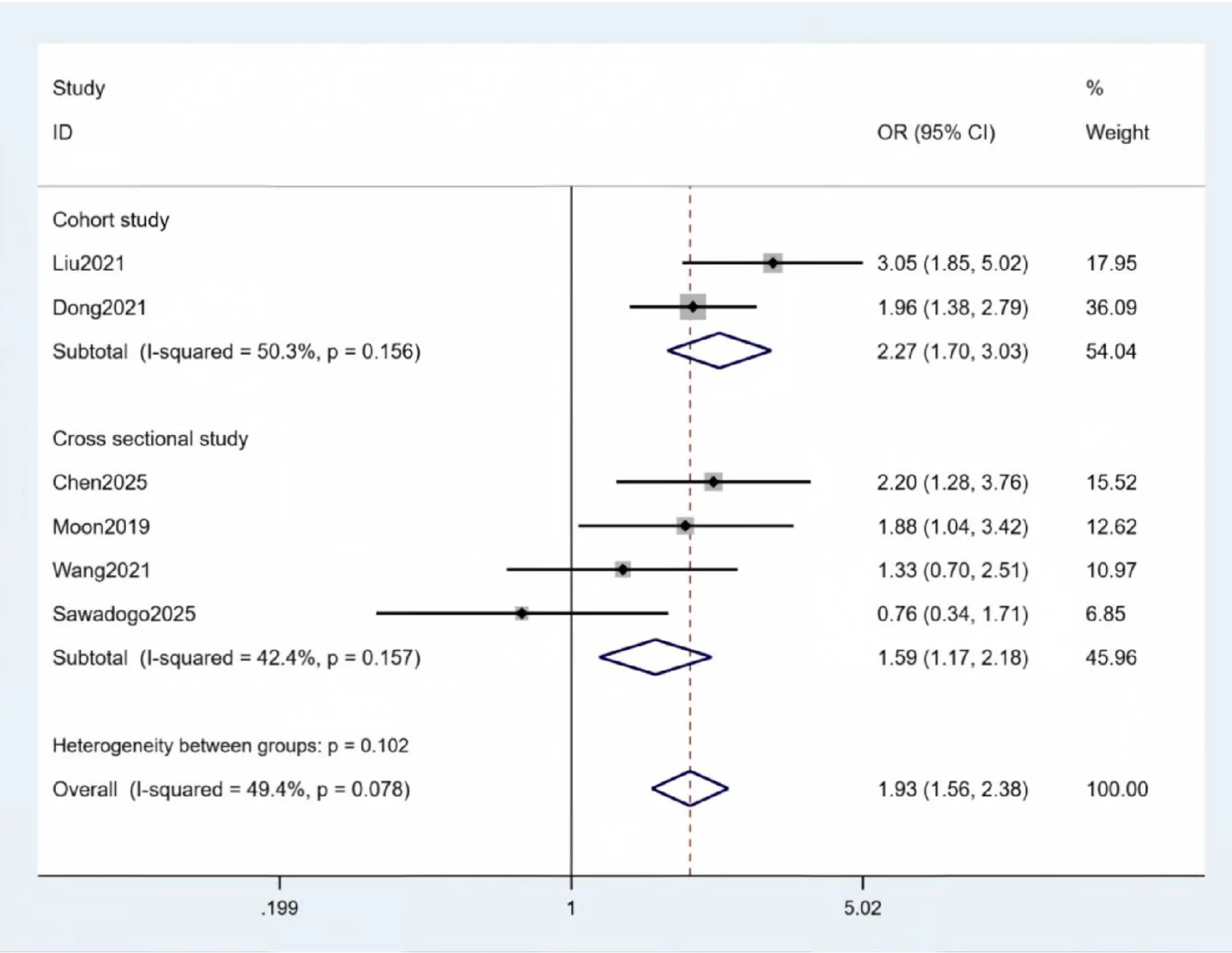
Forest plot of the association between short sleep duration and the risk of post-stroke depression, stratified by study design.

### 3.5 Publication bias and sensitivity analysis

Publication bias was assessed for the six studies on short sleep duration and the risk of post-stroke depression. The Begg test (*p* = 0.060 > 0.05) and Egger test (*p* = 0.239 > 0.05) showed no significant asymmetry, suggesting no clear evidence of publication bias. Sensitivity analysis indicated that the pooled results were stable and reliable, and that the included studies were reasonably representative (Figure 6).

**Figure 6:**
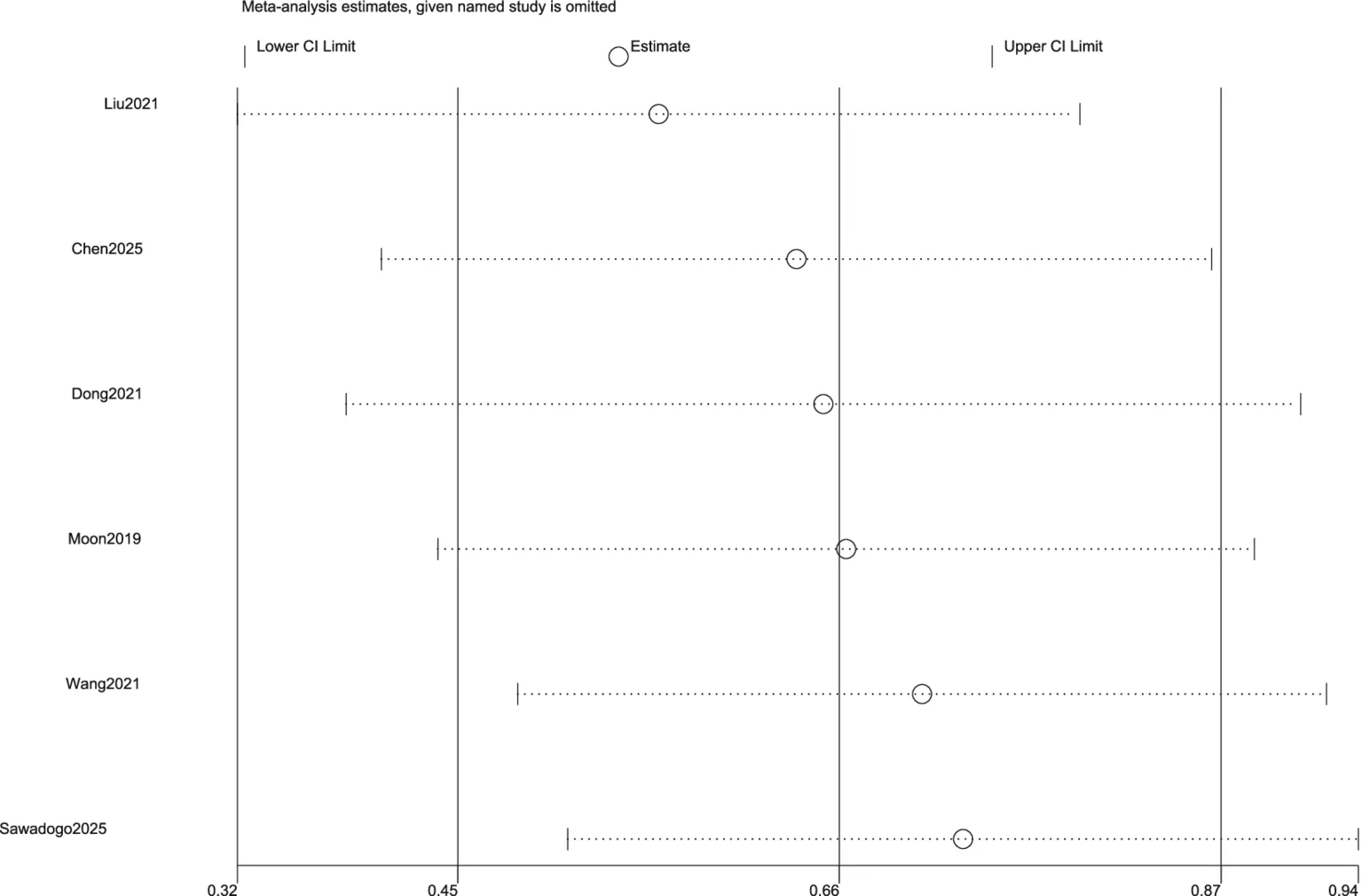
Sensitivity analysis 1.

Publication bias was assessed for the five studies on long sleep duration and the risk of post-stroke depression. The Begg test (*p* = 0.806 > 0.05) and Egger test (*p* = 0.235 > 0.05) showed no significant asymmetry, suggesting no clear evidence of publication bias. Sensitivity analysis indicated that the pooled results were stable and reliable, and that the included studies were reasonably representative (Figure 7).

**Figure 7:**
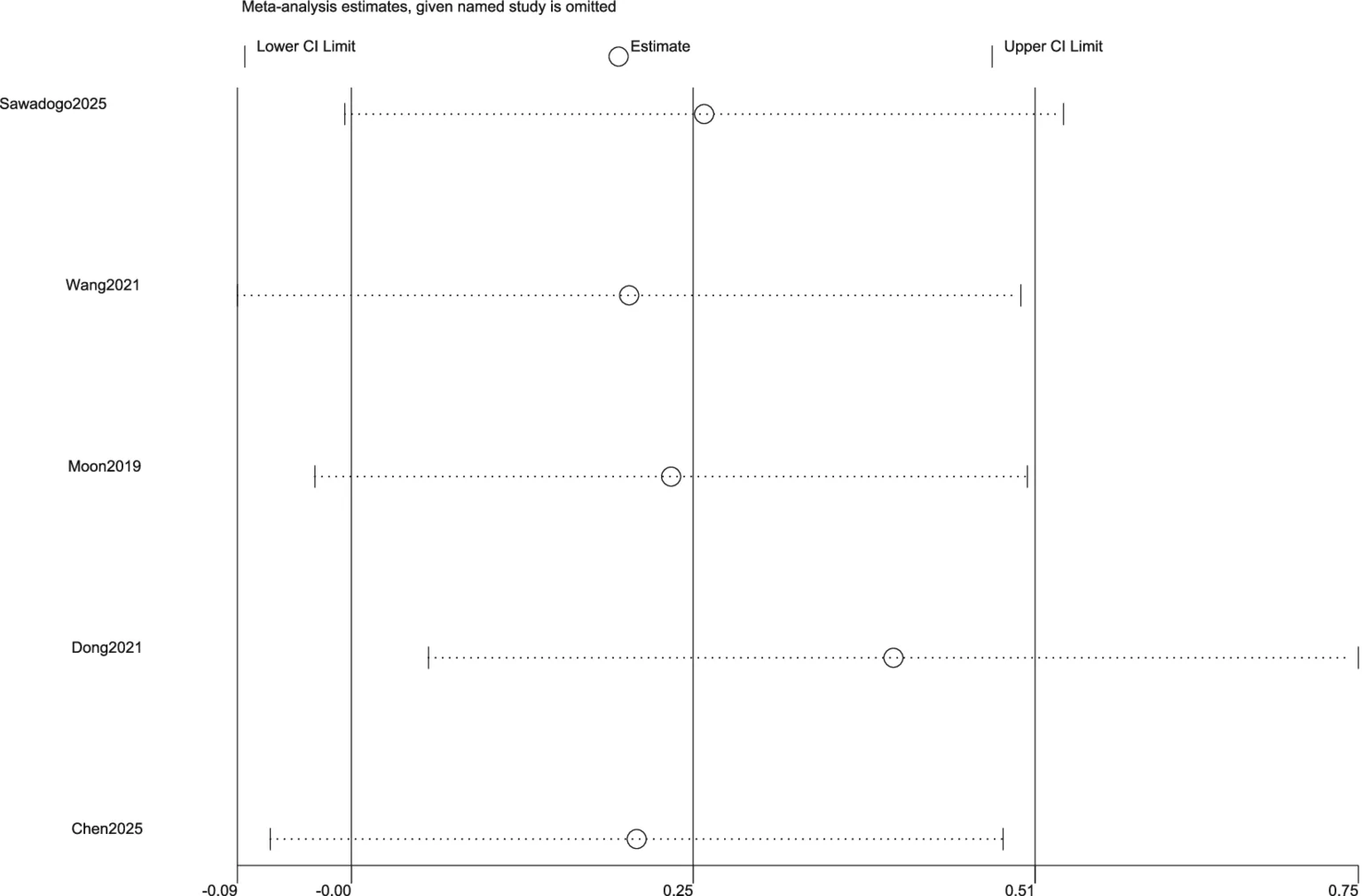
Sensitivity analysis 2.

## 4 Discussion

A recent meta-analysis published in 2025 investigated the association between post-stroke sleep disorders and post-stroke depression (PSD) (26); however, it treated sleep disorders as a broad exposure and did not specifically assess the relationship between sleep duration and the risk of PSD. Therefore, the present study included six relevant studies to investigate the association between different sleep durations and the risk of post-stroke depression. Meta-analytic results indicated that sleep duration was associated with the risk of post-stroke depression, and short sleep duration increased the risk of PSD. Accordingly, optimizing sleep duration is critical for the prevention of post-stroke depression.

Due to variations in region, culture and ethnicity, inconsistent definitions for short, long and normal sleep duration existed among individual studies. For studies presenting multiple sleep duration categories, we extracted effect sizes for the shortest and longest sleep groups. Our findings revealed that short sleep duration was associated with an increased risk of PSD, in line with the results of Liu F (27). Liu F’s study revealed that participants sleeping 5–6 h or less than 5 h face a higher risk of post-stroke depression compared to those sleeping over 7 h. The mechanism association between sleep duration to post-stroke depression risk remains under investigation. Research suggests that short sleep duration can disrupt the functions of the prefrontal cortex, anterior cingulate cortex, amygdala, and striatum, affecting emotional regulation and control, which leads to negative emotions (28). Additionally, it can disturb the brain’s reward system, elevate cortisol levels, cause an imbalance in the hypothalamic–pituitary–adrenal (HPA) axis, and lead to hippocampal neuron degeneration, ultimately resulting in depression (29). It may also heighten the inflammatory response by increasing levels of tumor necrosis factor-α (TNF-α), interleukin 6 (IL-6), and C-reactive protein (CRP), thereby worsening anxiety and depression (30). No significant association was observed between long sleep duration and post-stroke depression (PSD), consistent with findings by Chen (31). Notably, several previous studies have reported contradictory results, indicating that prolonged sleep duration may increase the risk of post-stroke depression. We believe that although patients with post-stroke depression had longer sleep duration in these studies, their sleep quality was poor, manifesting as frequent awakenings or difficulty falling asleep, which may also negatively affect mood (32). Furthermore, stroke patients often require prolonged bed rest due to physical dysfunction and neurological impairment. In this context, total bed rest time is likely to be misclassified as actual sleep duration (33). Failure to distinguish actual sleep time from bed rest time, as well as high-quality continuous sleep from fragmented sleep, may lead to the overestimation of true sleep duration. Such measurement bias is particularly prominent in the long-sleep subgroup and may obscure the potential association between prolonged sleep duration and PSD. Future studies with larger sample sizes and more original data focusing on long-sleep populations are warranted to clarify the genuine association between extended sleep duration and PSD risk among stroke survivors. Subgroup analysis by region shows that short sleep duration is a risk factor for post-stroke depression in both Asian and United States populations, indicating it is not unique to specific regions. This suggests that, in both Asian and US populations, sleep duration should be considered when assessing depression risk in stroke patients (14), and early sleep intervention should be implemented for those with short sleep to mitigate their depression risk. Subgroup analysis by study design reveals that both cohort and cross-sectional studies demonstrate that short sleep duration is associated with increased risk of post-stroke depression, with cohort studies demonstrating a stronger association. This may be because cohort studies can dynamically track changes during follow-up, providing more reliable evidence (34). Future studies may preferentially adopt a cohort study design; strict control of follow-up procedures and confounders is required to reduce heterogeneity, enhance the reliability of the results, and yield more stable effect estimates.

## 5 Limitations and prospects

Firstly, sleep duration in most included studies was assessed via self-reported measures, which are inherently subjective and susceptible to recall bias, potentially introducing bias into the pooled results. Future research should employ objective sleep monitoring devices (e.g., actigraphy, polysomnography) for the accurate measurement of sleep duration. Secondly, the number of included studies was limited, and most of them were cross-sectional. Further investigations are warranted to incorporate more prospective cohort studies to strengthen the reliability of the conclusions. Third, the included studies employed different assessment tools for outcome measures. Several of these scales had not undergone validation, which may have influenced the pooled effect size. Lastly, the definitions and cut-off values for short and long sleep in the included original studies were inconsistent, which may introduce heterogeneity and affect the stability of pooled results.

## 6 Conclusion

Sleep duration is significantly associated with the risk of post-stroke depression, and short sleep duration increases the risk of developing post-stroke depression; therefore, greater awareness of disease prevention is needed. Future strategies for early prevention of post-stroke depression should consider sleep as a critical factor. Ensuring stroke patients receive adequate sleep (7–9 h) daily is essential. In clinical settings, assessing sleep duration should be part of routine screening for stroke patients to identify those at high risk due to insufficient sleep. Implementing targeted sleep health guidance and emotional monitoring can help prevent post-stroke depression, reduce its incidence, and enhance quality of life.

## Data Availability

The original contributions presented in the study are included in the article/supplementary material, further inquiries can be directed to the corresponding author.
